# Survey of mosquito-borne flaviviruses in the Cuitzmala River Basin, Mexico: do they circulate in rodents and bats?

**DOI:** 10.1186/s41182-018-0117-6

**Published:** 2018-10-24

**Authors:** Jesús Sotomayor-Bonilla, Omar García-Suárez, Nohemí Cigarroa-Toledo, Rosa C. Cetina-Trejo, Ana C. Espinosa-García, Rosa E. Sarmiento-Silva, Carlos Machain-Williams, Diego Santiago-Alarcón, Marisa Mazari-Hiriart, Gerardo Suzán

**Affiliations:** 1 Programa de Maestría y Doctorado en Ciencias de la Producción y de la Salud Animal, Universidad Nacional Autónoma de México, Circuito interior s/n, Ciudad Universitaria, Coyoacán, Mexico City Mexico; 2 Laboratorio de Ecología de Enfermedades y Una Salud, Departamento de Etología, Fauna Silvestre y Animales de Laboratorio, Facultad de Medicina Veterinaria y Zootecnia, Universidad Nacional Autónoma de México, Circuito Interior s/n, Ciudad Universitaria, Coyoacán, Mexico City Mexico; 3 Asociación Mexicana de Medicina de la Conservación Kalaan Kab, A.C., Ciclistas 63 Col. Country Club, Coyoacán, Mexico City Mexico; 4 Laboratorio Nacional de Ciencias de la Sostenibilidad, Instituto de Ecología,, Universidad Nacional Autónoma de México, Circuito Exterior s/n anexo Jardín Botánico exterior, Ciudad Universitaria, Coyoacán, Mexico City Mexico; 5 Laboratorio de Arbovirología, Centro de Investigaciones Regional “Hideyo Noguchi”, Universidad Autónoma de Yucatán , Mérida, Yucatán, Mexico; 6 Departamento de Microbiología e Inmunología, Facultad de Medicina Veterinaria y Zootecnia, Universidad Nacional Autónoma de México, Circuito interior s/n, Ciudad Universitaria, Coyoacán, Mexico City México; 7 Red de Biología y Conservación de Vertebrados, Instituto de Ecología AC, Carretera Antigua a Coatepec 351, Xalapa, Veracruz México

**Keywords:** Arboviruses, Rodent, Bat, Dengue, West Nile virus, Host-virus interaction

## Abstract

**Background:**

RNA viruses commonly infect bats and rodents, including mosquito-borne flaviviruses (MBFV) that affect human and animal health. Serological evidence suggests past interactions between these two mammalian orders with dengue viruses (DENV), West Nile virus (WNV), and yellow fever virus (YFV). Although in Mexico there are reports of these viruses in both host groups, we know little about their endemic cycles or persistence in time and space.

**Methods:**

Rodents and bats were captured at the Cuitzmala River Basin on the Pacific coast of Jalisco state, Mexico, where MBFV, such as DENV, have been reported in both humans and bats. Samples were taken during January, June, and October 2014, at locations adjacent to the river. Tissue samples were collected from both bats and rodents and serum samples from rodents only. Highly sensitive serological and molecular assays were used to search for current and past evidence of viral circulation.

**Results:**

One thousand nine hundred forty-eight individuals were captured belonging to 21 bat and 14 rodent species. Seven hundred sixty-nine liver and 764 spleen samples were analysed by means of a specific molecular protocol used to detect flaviviruses. Additionally, 708 serum samples from rodents were examined in order to demonstrate previous exposure to dengue virus serotype 2 (which circulates in the region). There were no positive results with any diagnostic test.

**Discussion:**

To our knowledge, this is the first survey of rodents and only the second survey of bats from the Pacific Coast of Mexico in a search for MBFV. We obtained negative results from all samples. We validated our laboratory tests with negative and positive controls. Our findings are consistent with other empirical and experimental studies in which these mammalian hosts may not replicate mosquito-borne flaviviruses or present low prevalence.

**Conclusions:**

True-negative results are essential for the construction of distribution models and are necessary to identify potential areas at risk. Negative results should not be interpreted as the local absence of MBFV in the region. On the contrary, we need to establish a long-term surveillance programme to find MBFV presence in the mosquito trophic networks, identifying the potential role of rodents and bats in viral dynamics.

## Background

Mosquito-borne flaviviruses (MBFV; genus *Flavivirus*, family *Flaviviridae*) include some of the major emerging and re-emerging RNA viruses worldwide, such as dengue virus (DENV), Zika virus (ZIKV), and West Nile virus (WNV) [1]. MBFV affect millions of humans, domestic animals, and wildlife [1]. They are transmitted by many mosquito species that feed on a diverse array of vertebrate hosts. Thus, recognizing potential hosts within transmission cycles is crucial in order to predict and prevent an eventual MBFV emergence. Recently, some MBFV have expanded their continental distribution (ZIKV and Usutu virus) as a result of trading, travel, and the expansion of human populations (DENV and Rocio virus) [2], while epizootic and enzootic transmission cycles have proven to be very dynamic in the face of current global changes [1].

Rodent and bat communities are highly diverse, abundant, and accessible to capture in sufficient numbers to permit ecological and epidemiological studies [3]. Both host groups inhabit nearly all environments. Many species are well-adapted to human activities and harbour a high diversity of zoonotic pathogens, including MBFV [3]. In the USA, WNV and St. Louis encephalitis virus (SLEV) have been isolated from rodents (*Sciurus carolinensis*) and Mexican free-tailed bats (*Tadarida brasiliensis*), respectively [4, 5]. There are also reports of DENV in Neotropical rodents and bats inhabiting remote sylvatic, rural, and urban areas [6–9]. However, the role of these hosts in MBFV transmission cycles remains unknown. This dearth of knowledge limits our opportunities to prevent future viral emergence events. Here, we show the results of a molecular and serological survey of MBFV in Neotropical rodents and bats in an area where DENV outbreaks are endemic in human populations.

## Methods

**Fig. 1:**
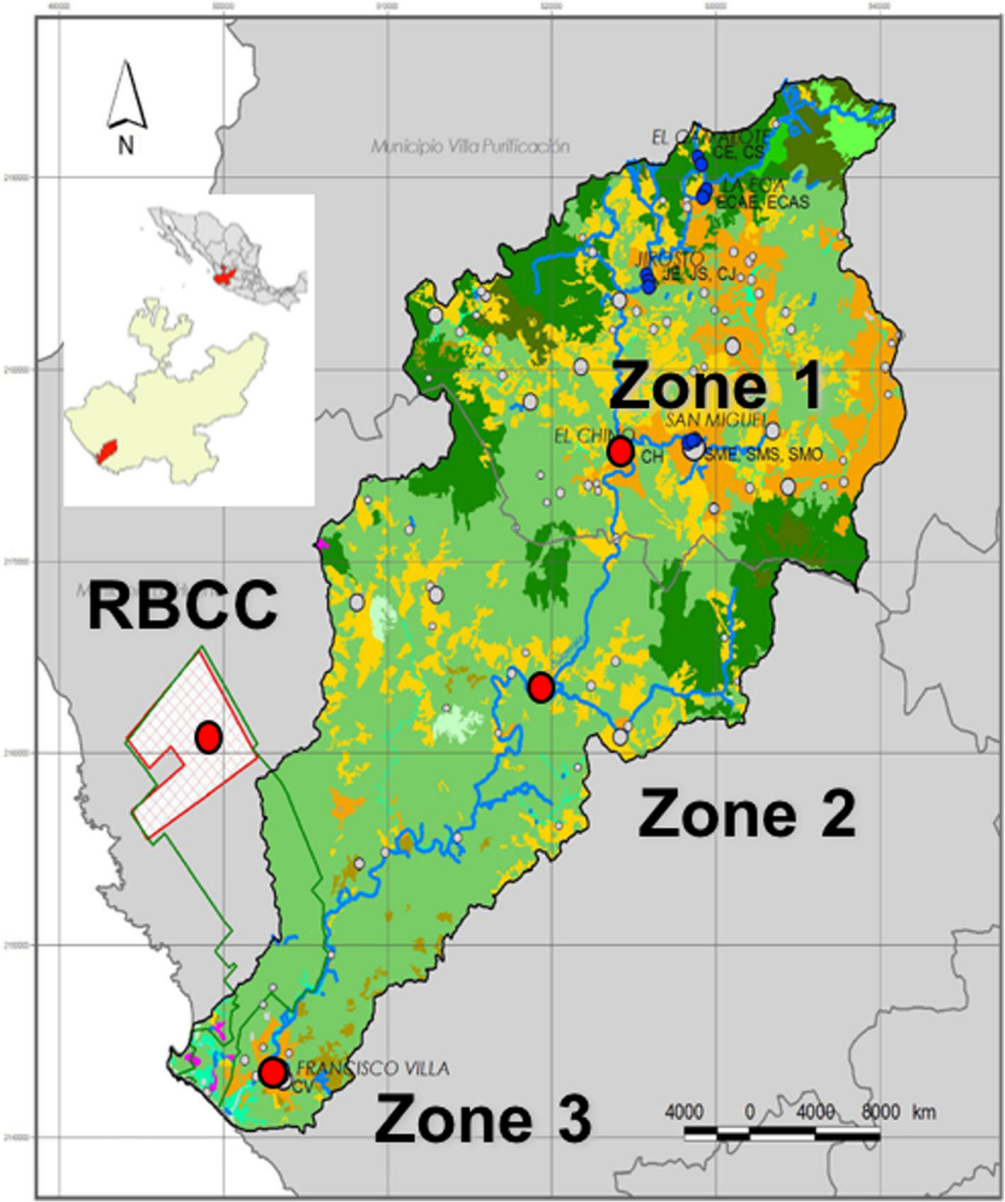
Map of study sites (red dots) in the Cuitzmala River Basin, Jalisco, Mexico

The study area was the Cuitzmala River Basin, located on the Pacific coast of Jalisco state in Mexico (Fig. 1). Deciduous and sub-deciduous dry tropical forest (DTF), crops, and pastures dominate the landscape. The Biosphere Reserve Chamela-Cuixmala (RBCC) is located in the lower section of the basin. There, health services in urban and rural settlements are deficient, with human cases of DENV being typical [10]. Some data also suggests DENV infection in bats [11].

We selected three study sites adjacent to the river to capture mammals: zone 1, from 2400 to 1000 m above sea level (masl); zone 2, from 1000 to 200 masl; and zone 3, from 200 masl), as well as two other sites in the RBCC. We visited each site in January, June, and October 2014. We captured rodents at three sample points per site (only one in the RBCC) separated by 500 m each, employing 100 Sherman traps per location, baited with a mixture of oats and vanilla essence. Traps were active during three consecutive nights and reviewed every morning. We captured bats at two sample points per site in riparian locations. For each sample point, we used five mist nets (9 × 3 m) that were opened after sunset and remained active for four consecutive hours. We used local field guides for taxonomic identification [12].

A representative number of rodents and bats was anaesthetized and euthanised with isoflurane and necropsied following international guidelines [13]. Liver and spleen samples from bats and rodents were immediately frozen in liquid nitrogen to conduct molecular tests. We extracted RNA from individual tissues using Trizol Reagent (Invitrogen Corp., Carlsbad, CA, USA), following the manufacturer’s instructions. The RNA of some samples was quantified in order to guarantee the viability of the samples and the procedure. We synthesised complementary DNA (cDNA) from all samples using a commercial kit (Thermo Scientific^R^, Waltham, MA, USA) and then made pools of ten individual samples to perform the semi-nested pan-flavivirus PCR protocol described by Scaramozzino et al. [14], using primers cFD2 (GTGTCCCAGCCGGCGGTGTCATCAGC), MAMD (AACATGATGGGRAARAGRGARAA), and FS778 (AARGGHAGYMCDGCHATHTGGT). This test detects a conserved region of the NS5 gene of the MBFV genus. It is highly sensitive and detects flaviviruses at a minimum of 200 infectious doses ml^−1^, including DENV, WNV, YFV, and ZIKV, as well as unknown flaviviruses [14]. We used DENV2-RNA provided by the Arbovirology Laboratory (Universidad Autónoma de Yucatán) as a positive control and nuclease-free water as negative control. PCR products were stained with GelRed (Biotium, Inc., CA, USA) and visualised in 2% agarose gels.

We collected rodent blood samples from the retro-orbital sinus using Nobuto strips and stored them at room temperature until laboratory processing. Nobuto strips were eluted by cutting the blood-absorbing portion, placing them in a tube containing 400 μl of phosphate-buffered saline solution. Eluates were then transferred to sterile tubes. Proteins of some samples were quantified in order to guarantee the viability of the samples and the procedure.

We performed an enzyme-linked immunosorbent assay (ELISA) test (as described in Reference [15]) to look for evidence of past infection by DENV-2, as a representative MBFV. As antigen, we propagated an Asian/American genotype DENV-2 in C6/36 cells [16], and we used MAb6b6C-1 (specific for the envelope (E) protein of MBFV) as the primary antibody. We calculated seropositivity as described in reference [17], a test that has been widely used in systematic flavivirus surveys in wildlife [17].

## Results

Although 1948 individuals belonging to 21 bat and 14 rodent species were captured, we were only able to screen by pan-flavivirus PCR 1569 samples from 13 rodent and 12 bat species (796 liver and 773 spleen samples; Table 1). No MBFV RNA was detected. We also examined 708 rodent serum samples for evidence of past DENV infection. All samples were negative. Negative and positive controls were as expected, supporting the quality of the detection procedures.

**Table 1:** Bat and rodent species, and the number of serum, liver, and spleen samples tested

	Total ELISA for DENV-2′ serum samples	Total Pan-Flavivirus PCR liver samples	Total Pan-Flavivirus PCR spleen samples
Bat species			
*Artibeus jamaicensis*		6	9
*Artibeus phaeotis*		1	1
*Artibeus watsoni*		1	
*Centurio senex*		1	1
*Choeroniscus godmani*		1	1
*Desmodus rotundus*		55	51
*Glossophaga commissarisi*		3	2
*Glossophaga soricina*		4	4
*Leptonycteris curasoae*		1	1
*Pteronotus parnellii*		3	1
*Sturnira lilium*		3	4
*Sturnira ludovici*		4	2
*Rodent species*			
*Baiomys musculus*	161	162	155
*Hodomys alleni*	8	3	3
*Liomys pictus*	199	193	194
*Mus musculus*	8	22	23
*Oryzomys couesi*	50	84	81
*Oryzomys melanotis*	61	57	55
*Osgoodomys banderanus*	73	37	34
*Peromyscus perfulvus*	59	46	47
*Rattus norvegicus*	2	4	4
*Rattus rattus*	2	14	14
*Reithrodontomys fulvescens*	29	31	28
*Sigmodon alleni*	14	15	14
*Sigmodon mascotensis*	38	45	44
*Spermophilus annulatus*	4		
*Total*	708	713	696
*Total sample effort*	708	796	773

## Discussion

Our study is the first rodent and second bat survey searching for MBFV in the Cuitzmala River Basin region [11]. We tested a representative sample of all individuals, including 10.79% in bat captured and 60.47% in rodents captured. In all cases, the results were negatives. Our findings are consistent with other empirical and experimental studies [17–20]. For example, Cabrera-Romo et al. [18] found no evidence of DENV infection in 240 Mexican bats.

There is no molecular evidence of MBFV, or evidence of antibody production against DENV-2, in wild rodents from Mexico, although there are reports of DENV in urban rodents (*Mus musculus* and *Rattus rattus*) [8, 21], demonstrating that environmental heterogeneity may directly affect MBFV circulation in these hosts. We found no molecular evidence of MBFVs in rodent samples or evidence of antibody production against DENV. In other countries, low prevalence of MBFV in wild rodents has been reported [17, 20]. However, sample viability is diminished by the preservation procedure and the time that has passed until serological testing. DENV sequences have been retrieved from different rodent species (*Oryzomys capito*, *Proechimys cuvieri*, *Mesomys hispidus*, and *Zigodontomys brevicauda*) from French Guiana [9]. Also, in the USA, SLEV and WNV have been reported in cricetid (*Sigmodon hispidus*, *Oryzomys palustris*) and sciurid rodents (*Tamiasciurus hudsonicus*, *Tamias striatus*) [4, 22]. None of the wild rodent species tested in this study were included in past surveys [8, 21].

Regarding bats, we included species previously reported as positive for DENV near our study areas, such as *Artibeus jamaicensis*, *Sturnira lilium*, *Pteronotus parnellii*, and *Desmodus rotundus* [11]. However, we found no molecular evidence of MBFV. There are reports of DENV, WNV, and SLEV in 26 Neotropical bat species inhabiting Southern Mexico, including frugivorous (*Carollia* spp.), insectivorous (*Molossus* spp.), nectarivorous (*Glossophaga soricina*), and haematophagous (*Desmodus rotundus*) bats [6, 9, 23, 24]. Interestingly, viral sequences and isolates have been retrieved from different types of tissues [6, 7, 9]. For example, DENV sequences were obtained from liver samples of frugivorous bats and intestine samples of insectivorous bats [6, 9], while SLEV was isolated from the saliva of Mexican free-tailed bats [5]. Our negative results suggest an important spatio-temporal variability of MBFV in the region, which might also be expected at other locations, in particular, those where suitable environmental conditions for vectors vary across the year.

## Conclusion

Bats and rodents deserve more attention as potential alternative host species, reservoirs, and dead-end hosts of MBFV (e.g. DENV) [6]. Negative results do not exclude a potential infection state amongst hosts nor the potential circulation of MBFV in the region. Thus, we should not underestimate the existence of yet undiscovered sylvatic and sporadic cycles that may involve host communities connected by dispersal, and which can maintain cycles that would otherwise become extinct in individual species at the local level. Recognizing these viruses in nature is not straightforward, given the vast variety of elements that influence their transmission [25]. We suggest that (1) rodents and bats do not always participate in MBFV transmission cycles within the region, (2) that tested species may not generate sufficient viremia to be detected by RT-PCR protocols, (3) that the presence of IgM in serum samples could not be detected by competitive ELISA tests, and (4) that our sampling period did not match space-time with infected hosts. To rule out the role of these communities in MBFV transmission cycles, it is necessary to implement long-term studies, to increase the number and type of samples tested and to use more advanced molecular and serological diagnostic tests (e.g. microarrays or plaque reduction neutralization tests). It is also essential to carry out experimental studies to further determine the role of the hosts. Finally, it is crucial to simultaneously study feeding preferences and viral circulation in the regional mosquito community.

## Abbreviations

DENV: Dengue virus
DNA: Deoxyribonucleic acid
E: Envelope
ELISA: Enzyme-linked immunosorbent assay
MBFV: Mosquito-borne flavivirus
PCR: Polymerase chain reaction
RBCC: Biosphere Reserve Chamela-Cuixmala
RNA: Ribonucleic acid
SLEV: St. Louis encephalitis virus
TDF: Tropical dry forest
WNV: West Nile virus
YFV: Yellow fever virus
ZIKV: Zika virus
